# A Pragmatic Approach to Guide Implementation Evaluation Research: Strategy Mapping for Complex Interventions

**DOI:** 10.3389/fpubh.2018.00134

**Published:** 2018-05-18

**Authors:** Alexis K. Huynh, Alison B. Hamilton, Melissa M. Farmer, Bevanne Bean-Mayberry, Shannon Wiltsey Stirman, Tannaz Moin, Erin P. Finley

**Affiliations:** ^1^VA Greater Los Angeles HSR&D Center for the Study of Healthcare Innovation, Implementation and Policy, Los Angeles, CA, United States; ^2^David Geffen School of Medicine, University of California Los Angeles, Los Angeles, CA, United States; ^3^Department of Psychiatry and Behavioral Sciences, Stanford University, Palo Alto, CA, United States; ^4^VA Palo Alto Healthcare System, Menlo Park, CA, United States; ^5^South Texas Veterans Healthcare System, San Antonio, TX, United States; ^6^UT Health Science Center, San Antonio, TX, United States

**Keywords:** implementation strategies, strategy mapping, complex interventions, implementation blueprint, evaluation

## Abstract

**Introduction:**

Greater specification of implementation strategies is a challenge for implementation science, but there is little guidance for delineating the use of multiple strategies involved in complex interventions. The Cardiovascular (CV) Toolkit project entails implementation of a toolkit designed to reduce CV risk by increasing women’s engagement in appropriate services. The CV Toolkit project follows an enhanced version of Replicating Effective Programs (REP), an evidence-based implementation strategy, to implement the CV Toolkit across four phases: pre-conditions, pre-implementation, implementation, and maintenance and evolution. Our current objective is to describe a method for mapping implementation strategies used in real time as part of the CV Toolkit project. This method supports description of the timing and content of bundled strategies and provides a structured process for developing a plan for implementation evaluation.

**Methods:**

We conducted a process of strategy mapping to apply Proctor and colleagues’ rubric for specification of implementation strategies, constructing a matrix in which we identified each implementation strategy, its conceptual group, and the corresponding REP phase(s) in which it occurs. For each strategy, we also specified the actors involved, actions undertaken, action targets, dose of the implementation strategy, and anticipated outcome addressed. We iteratively refined the matrix with the implementation team, including use of simulation to provide initial validation.

**Results:**

Mapping revealed patterns in the timing of implementation strategies within REP phases. Most implementation strategies involving the development of stakeholder interrelationships and training and educating stakeholders were introduced during the pre-conditions or pre-implementation phases. Strategies introduced in the maintenance and evolution phase emphasized communication, re-examination, and audit and feedback. In addition to its value for producing valid and reliable process evaluation data, mapping implementation strategies has informed development of a pragmatic blueprint for implementation and longitudinal analyses and evaluation activities.

**Discussion:**

We update recent recommendations on specification of implementation strategies by considering the implications for multi-strategy frameworks and propose an approach for mapping the use of implementation strategies within complex, multi-level interventions, in support of rigorous evaluation. Developing pragmatic tools to aid in operationalizing the conduct of implementation and evaluation activities is essential to enacting sound implementation research.

## Background

With rapid growth in the field of implementation science has come increasing complexity in the way that studies are planned and executed. Evidence-based interventions to improve the quality of care are frequently multi-component, comprised of, for example, both patient- and provider-facing elements (1). Implementation efforts are often large-scale and likely to be conducted across multiple sites simultaneously, each of which may have its own unique characteristics, needs, and resources (2). There is a growing array of implementation strategies—“methods or techniques used to enhance the adoption, implementation, and sustainability of a clinical practice or program” (3)—available to address the varied needs of different sites. Correspondingly, the use of implementation strategies has become increasingly sophisticated, with a growing number of efforts using a combination of strategies to target multiple levels of an organization (e.g., providers, middle managers, and high-level administrators).

There has been an increasing call for implementation research studies to describe their use of implementation strategies with greater specificity and precision, with two primary goals: replication and evaluation (4). At its most basic, this call for greater precision in the description of implementation strategies seeks to increase our ability to identify and replicate strategies that are effective in supporting adoption, scale-up, and spread of best practices in health care (4). Precise specification of how implementation strategies are used allows for greater ability to evaluate their effectiveness, understand potential mechanisms of action, and identify areas for improvement, thereby contributing to rapid evolution of the knowledge base in implementation science (3, 4). It is well recognized that there is poor replication of clinical interventions (5), and we often see the same phenomenon in implementation studies, with initially promising strategies failing to show impact in later efforts (6–8). Consequently, many implementation studies occur as isolated events, and the opportunity to build incrementally toward a knowledge base for effective implementation is compromised.

In response to this concern, a growing literature has called for standardization in implementation reporting, encouraging use of a common language for naming and defining strategies and describing their functional components (3, 9–11). Powell and colleagues (9) have done much to support this effort by developing a compilation of 73 discrete implementation strategies through a process of expert review. Waltz and colleagues (10) proposed a taxonomy for organizing those 73 strategies into nine overarching conceptual categories reflecting their core goals and approaches (e.g., involving stakeholders, education, etc.). Proctor and colleagues (3) have offered guidelines for the specification of implementation strategies, recommending that each implementation strategy be described in terms of seven domains: the actors involved, actions undertaken, action targets, timing or temporality, dose, implementation outcomes, and theoretical justification.

The development of these rubrics for defining and specifying implementation strategies has resulted in a significant change in how implementation research is described, and the level of information available to support understanding and interpretation of findings. For example, Bunger and colleagues (11) developed a method for using activity logs as part of a multi-component effort to improve children’s access to behavioral health services. Use of these detailed logs facilitated the identification of discrete strategies enacted over time, while also supporting documentation of the implementation activities, intent, duration, and actors involved. This documentation allowed for more precise estimation of the effort involved. Gold and colleagues (12) engaged in similar description of implementation strategies operationalized as part of a diabetes quality improvement intervention occurring in commercial and community healthcare settings. They found that, while the strategies utilized and outcome observed were constant across settings, specific components of the strategies used—including actor, action, temporality, and dose—were adapted to fit local contexts, thus underscoring the importance of flexibility in implementation (12). Most recently, Boyd and colleagues (13) coded implementation team meetings to characterize implementation strategies. They identified six categories of strategies: quality management, restructuring, communication, education, planning, and financing, including one (communication) that had not been identified as such in previous taxonomies. In preliminary analyses, financing was associated with greater intervention fidelity. In another recent study, Rogal and colleagues used an electronic survey to assess use of specific strategies in implementation of evidence-based hepatitis C treatment (14). In doing so, they were able to identify 28 strategies that were significantly associated with initiation of evidence-based hepatitis C treatment, including use of data warehousing techniques and intervening with patients. Collectively, these studies have been pioneering in their use of the shared language offered by Proctor and colleagues (3) to achieve consistent reporting in implementation research; they point the way forward for future efforts.

Nonetheless, movement toward greater specification of individual implementation strategies raises challenges, particularly related to reporting on the kind of complex interventions integrating multiple strategies that are increasingly the norm. The work by Boyd and colleagues identified 39 unique strategies for each site in their study (6 sites total), while Bunger and colleagues identified 45 unique strategies in their implementation activities (11, 13). In addition, implementation is frequently a multi-phased process, requiring preparatory work, implementation launch, as well as post-implementation activities aimed at increasing reach, adoption, or sustainment (15). And yet most implementation evaluations focus on a single phase of the process, most commonly implementation. This allows for focused examination of core activities and lessons learned, as in a recent study of factors associated with uptake of an evidence-based exercise group for seniors (16), but may constrain the information available on how strategies were used over the full course of implementation (17). This has limited the amount of empirical data available on how the timing of specific strategies, or the sequence in which they are rolled out, may impact the success of implementation. In one novel study attempting to tackle this problem, Yakovchenko and colleagues conducted qualitative comparative analysis of strategies, and identified specific strategy combinations linked to high levels of treatment initiation (18). The authors were unable, however, to discern whether these findings were impacted by the timing or sequence of strategies (18). Similarly, although a handful of studies have examined implementation across multiple phases (11, 13, 15, 19), few have provided significant detail regarding when and how implementation strategies were deployed (20).

**Figure 1:**
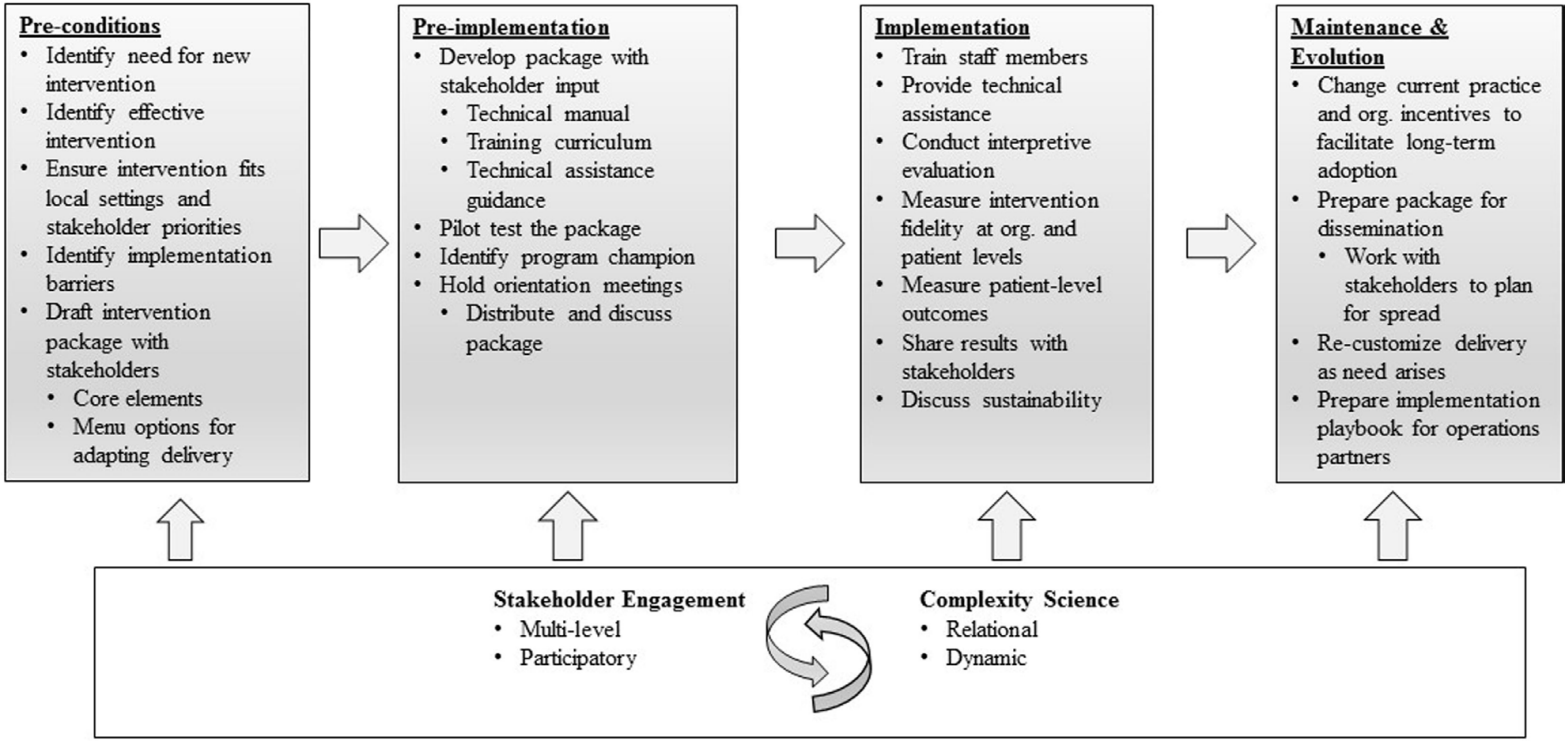
Replicating Effective Programs Implementation Strategy*. Enhanced with stakeholder engagement and complexity science. *Adapted from Ref. (21, 22).

The question of how best to document and describe implementation strategies in multi-phase work, therefore, remains salient. In the implementation research described here, we draw upon the Replicating Effective Programs (REP) framework (21) (Figure 1), which functions as an evidence-based roadmap for the implementation of interventions by outlining implementation strategies to be employed across four phases: pre-conditions, pre-implementation, implementation, and maintenance and evolution (21, 22). During the pre-conditions and pre-implementation phases, careful attention is paid to intervention packaging. In the implementation phase, attention is paid to training, technical assistance, and fidelity. And in the maintenance and evolution phase, emphasis is placed on planning and recustomizing for long-term sustainment and spread (22, 23). The “Enhancing Mental and Physical Health of Women Veterans through Engagement and Retention” (EMPOWER) Quality Enhancement Research Initiative (QUERI), funded by the U.S. Department of Veterans Affairs (VA), has undertaken a program of three studies making shared use of REP as an organizing framework (24).

Although the call for greater specificity in describing implementation strategies is important in advancing implementation science, we have found little guidance on how to apply Proctor and colleagues’ recommendations in the context of complex, multi-component interventions, on at least three fronts. First, there is the question of how to ensure all strategies are effectively identified for reporting, given that frameworks such as REP have not previously been described in a manner consistent with newer taxonomies and specification guidelines. Second, use of packaged frameworks such as REP raises questions regarding how to track strategies that may occur at multiple time points, occur in a particular sequence, and/or overlap with other strategies. Similarly, guidance is rarely provided regarding whether component strategies are essential or optional, or their suggested dose or intensity, making it difficult to assess the fidelity with which the framework was followed in resulting trials. Third, evaluating the impact of specific strategies can be difficult, given that implementation outcomes are likely to reflect the cumulative impact of strategies over time.

In addition, there is a practical challenge associated with operationalizing complex implementation efforts across multiple sites, in ensuring all activities necessary for both implementation and evaluation are occurring at the appropriate time and place. Development of a formal implementation blueprint has been identified as an implementation strategy unto itself, with the suggestion that a blueprint should include the implementation effort’s aim or purpose, intended scope, timeframe, milestones, and appropriate progress measures, and that it should be used and updated over time (9). But while excellent guidelines exist for intervention mapping in health promotion more generally (25), preparing an implementation research proposal (26) or manuscript (27), as well as describing the suggested components of an implementation plan (28), relatively little literature has described how to develop a practicable blueprint for use in organizing the many-tentacled process of implementation evaluation.

To address these concerns, we embarked on a prospective, formative, and iterative team-based process for mapping a multi-component implementation strategy, REP, to recommended taxonomies of implementation strategies. Our primary goal in doing so was to support more effective evaluation of overlapping and sequenced implementation strategies. We also sought to support the operationalization of a complex intervention, providing an implementation blueprint to outline activities and tasks at each phase, and the actors or point persons responsible for those activities. In the current paper, we describe this process alongside the method by which we used the resulting strategy matrix to support development of a formal evaluation plan for one of the EMPOWER studies, “Facilitating Cardiovascular Risk Screening and Risk Reduction in Women Veterans” (known as CV Toolkit), aimed at using a gender-tailored toolkit to reduce cardiovascular (CV) risk among women Veterans in VA primary care settings (24).

## Methods

### Implementation Study

The CV Toolkit is comprised of evidence-informed practices aimed at reducing CV risk among patients in primary care and tailored to meet the needs of women Veterans in the VA (Table 1). The CV Toolkit evolved in response to a need for consistent screening and documentation, increased CV risk reduction services and support for women Veterans in VA primary care. REP pre-conditions work leading up to the formal CV Toolkit study included obtaining input from national operations partners and clinical stakeholders regarding potential gaps in women Veterans’ CV risk assessment and care services (24). Pre-conditions work also included focus groups conducted by the study leads (BBM and MF) with primary care providers and women Veteran patients, who identified a variety of barriers and facilitators to effective CV risk management (24). The CV Toolkit was developed as a set of evidence-informed practices intended to address the needs identified by stakeholders and is centered around three specific items: patient education and self-screening of CV risks, provider documentation of CV risks in the electronic health record, and a facilitated group to help patients identify and set behavioral health goals [e.g., the Gateway to Healthy Living program (hereafter, Gateway)]. Gateway is a VA program first piloted in 2015 and now being implemented across VA nationwide, which focuses on motivating and supporting Veterans with chronic conditions such as CV disease or risk conditions to engage in services aimed at reducing their risk (29). Previous evaluation of patient experiences with Gateway suggest high rates of goal setting and linking patients to existing programs, as well as high satisfaction with the Gateway sessions (29). In addition, surveys of staff suggest that the Gateway program was perceived as “very helpful” in connecting Veterans to programs and resources (29).

**Table 1:** Summary of Cardiovascular (CV) Toolkit components.

Component	Purpose
Patient education and activation	
Information sheets, posters, brochures	Educate patients regarding CV risks
Opt-in/Opt-out step	Inform eligible patients can choose to participate in research component of project (surveys/interviews)
Patient self-report CV risk screener	Identify patients with any CV risks; Facilitate patient–provider communication regarding risk factors and appropriate health goals
CV risk computerized template	Identify and document patients with CV risks using template and using risk calculator, embedded within the electronic health record Track use of template by providers on unique patients Track patient–provider action step or goal
Gateway to healthy living facilitated group	Educate patients about options for healthy living; provide support in setting behavioral health goals and referrals to appropriate services Identify and track CV goal set by patient in group
Follow-up phone calls	Follow-up phone calls after Gateway attendance to assess progress with behavioral health goals Identify barriers to CV goal

The CV Toolkit provides a process for assessing women’s CV risk via a patient self-report risk screener, facilitates patient–provider communication and documentation of risk data via a provider-facing computer template embedded in the electronic medical record, and educates providers in shared decision-making and effective clinical action around risk reduction. Women are given the option of participating in women-only Gateway groups, which are tailored for women and focus on CV risk, offer patient education and activation, and serve as an entry point for patients to receive information, goal setting, and referral to other programs and services as needed [additional detail on this and other EMPOWER projects is available (24)].

Having been developed specifically to meet the needs of women Veterans in VA primary care, the CV Toolkit is currently being implemented at two VA facilities with moderately large comprehensive Women’s Health (WH) clinics, with two additional facilities slated for future implementation. Clinics are eligible if they have multiple primary care providers serving women patients (ideally 6 or more providers) and each provider has at least 100 unique women Veteran patients and at least 10% of their total patient panel is female. Implementation of the CV Toolkit is being evaluated using a non-randomized stepped wedge design to detect differences before and after implementation at each site; this design will also allow for comparisons across sites and providers as the toolkit is implemented (30). The objective of the current work was to develop a step-by-step blueprint operationalizing use of implementation strategies across the CV Toolkit rollout, with the primary goal of guiding evaluation.

### Overview/Setting

To develop a comprehensive map of fully specified implementation strategies included as part of the CV Toolkit project, and to link these strategies to our longitudinal evaluation plan, we followed a five-step process, as outlined below. Participants in the strategy mapping process included six team members with overlapping roles central to implementation (including a clinician-researcher who serves as a liaison with sites and provides education for clinicians), intervention (including a health promotion specialist charged with leading Gateway groups and serving as an external facilitator for sites), and evaluation (including experts in health services and implementation research, anthropology, sociology, and biostatistics).

The five-step process includes the following:

(1) *Study activity list generation*. We first developed a list of CV Toolkit activities as described in the approved human subjects’ protocol, using a previously defined method for treating study documents as primary texts for analysis (31). The CV Toolkit protocol, including the implementation plan, was developed in response to findings from pre-conditions work, and therefore built upon deep knowledge of the VA primary care context and the needs and gaps in care for both women Veterans and their primary care providers. From the beginning, project activities were planned in accordance with the enhanced REP framework, with specific tasks occurring in sequence over the pre-conditions, pre-implementation, implementation, and maintenance and evolution phases. The enhanced REP framework used by EMPOWER QUERI projects (24), building on the original REP framework (21), places more focus on participatory action within complex adaptive systems in VA clinical settings. Once initial activity lists had been generated by two team members (Alexis K. Huynh and Erin P. Finley), these lists were compared and areas of initial discrepancy were discussed with the CV Toolkit Co-Principal Investigators (Bevanne Bean-Mayberry and Melissa M. Farmer) to achieve consensus. The team then categorized each activity as occurring in support of (1) research goals, (2) intervention delivery, or (3) implementation.
(2) *Mapping study activities to implementation strategies*. Once we had identified all implementation-related activities defined in the protocol, we then mapped these where possible to corresponding implementation strategies, as defined in the Powell compilation (9). As in Step (1), mapping was conducted separately by two team members and then compared, with any discrepancies discussed to consensus with study Co-PIs and other members of the project team, including those providing clinical care in targeted sites and working within the Gateway program. In most cases, the match was clear. Nonetheless, some REP activities did not map to any of the compiled strategies (e.g., collecting data on the timing of implementation launch, which we determined to be a research activity rather than implementation activity), and were not included in the strategy matrix.
(3) *Specifying implementation strategies by REP phase and conceptual category*. Early in the mapping process, it became clear that certain implementation strategies—e.g., coalition building—were occurring at multiple timepoints over the course of the CV Toolkit study. We therefore took care to specify how and when each strategy would be operationalized during each of the relevant REP phases (see Table 2 for final version) (21). In addition, following Proctor’s recommendations for reporting on use of implementation strategies, we provided full description across each of the seven domains for each strategy, including the actors involved, actions undertaken, targets, dose of the implementation strategy, and anticipated outcomes (3). We also organized the strategies into broader conceptual categories, as proposed by Waltz et al. (10), to evaluate whether specific categories of effort (e.g., stakeholder engagement) emerged at different phases over the course of the study.
(4) *Iterative refining of implementation strategy mapping*. An initial matrix summarizing the above work was reviewed during a series of team meetings with CV Toolkit study Co-PIs and the overall EMPOWER QUERI PI (Alison B. Hamilton), who provided feedback clarifying the nature, sequence, and/or intent of implementation-related activities. The matrix and mapping process were also presented to larger combined groups of implementation agents and researchers, who offered helpful input regarding how to make the matrix as comprehensive and streamlined as possible. The strategy matrix was iteratively refined from these meetings, resulting in a final matrix (see Table 2) providing detailed description of each implementation strategy planned as part of CV Toolkit implementation. The final strategy matrix was reviewed and *validated* by the full project team, including members responsible for implementation of the CV Toolkit as well as those tasked with evaluation.
(5) *Developing an implementation blueprint*. The completed strategy matrix provided a clear step-by-step plan for rolling out implementation strategies to facilitate implementation of the Toolkit, complete with their timing, target, and outcomes. This allowed us to plan for appropriate evaluation of our enhanced REP strategy at each site and across sites. In evaluating the effectiveness of CV Toolkit implementation across this study, we aim to quantitatively assess adoption of three components of the intervention: (1) completion of the CV risk template in the electronic health record by the provider or member of the care team; (2) number of patients who attend the Gateway to Healthy Living facilitated groups and number of follow-up calls made to patients following Gateway attendance; and (3) patient referrals for services. Data captured by the CV risk computer template and other administrative data will be used to examine these outcomes for each provider at each site and will allow us to assess whether and how adoption varies as strategies are enacted over the course of implementation. It is anticipated that successful adoption of CV Toolkit will also impact patient–provider communication and patient experiences of and engagement with care. We are therefore collecting qualitative data regarding patients’ and providers’ experiences of and engagement with CV Toolkit implementation, including adoption, acceptability, feasibility, engagement, and satisfaction (32). We are also conducting reflective discussions with team members to aid in documenting when and how key implementation activities occur (33). Taken in sum, these data will be integrated to allow for process and summative evaluation (see Table 3), as described in the published protocol (24).

**Table 2:** Strategies facilitating actions implementing Cardiovascular (CV) Toolkit over time [by Replicating Effective Programs (REP) phase and month].

Strategy	Actions by REP phase and month															
REP phase	Pre-condition			Pre-implementation			Implementation							Maintenance and evolution		
				
Month	1	…	6	7	…	12	13	14	15	…	25	26	27	28	…	31
1. Conduct local needs assessment	1. Establish need for the intervention 2. Determine feasibility at local site															

2. Inform local opinion leaders				1. Discuss CV Toolkit with key stakeholders during site visits 2. Explain core elements and options for adapting delivery			1. Regular communication with opinion leaders throughout intervention (to learn from them what is working and inform them what is not working at other sites).									

3. Develop educational materials	1. Review and select patient and provider educational materials 2. Discuss educational needs of teams at sites			1. Further local tailoring of educational materials for each site												

4. Promote adaptability				1. Explain core elements and options for adapting delivery to key stakeholders during site visits			1. Interactive component							1. Collaborate with local teams to develop CV Toolkit Implementation Playbook		

5. Build a coalition	1. Work with national-level partners 2. Engage with selected sites 3. Orient and elicit feedback from key stakeholders during site visits 4. Conduct needs assessment			1. Identify local champions 2. Hold broader orientation meetings 3. Orientation and adaptation at sites										1. Report on findings 2. Review business case 3. Collaborate to plan for spread 4. Collaborate to re-tailor as needed with spread					

6. Conduct educational meetings	1. Discuss educational needs of teams at sites			1. Hold orientation meetings with broader clinic at each site to distribute and discuss CV Toolkit and assess educational needs			1. Use monthly reflection calls with site leads to discuss and address challenges in implementation						

7. Tailor strategies	1. Refine, program, test, and load computer template in CV Toolkit package 2. Explore local resources to further tailor to site			1. Refine, program, test, and load computer template in CV Toolkit package 2. Work with national-level partners to make program adjustments and further tailor for women Veterans 3. Tailor locally with training and technical assistance 4. Explore local resources to further tailor to site 5. Develop communication plan at each site			1. Collaborate with local health coaches to tailor Gateway to site and women Veterans 2. Explore local resources to further tailor to site							1. Modify CV Toolkit as needed to continue and disseminate 2. Recustomize Implementation Playbook as needed		

8. Provide local technical assistance	1. Discuss educational needs of teams at sites			1. Overall launch meeting and training 2. Train & and detail for each provider on the computer template 3. Further local tailoring with training and technical assistance of toolkit package for each site			1. Assess additional need for detailing, provider training, and technical assistance									

9. Involve executive boards	1. Work with national-level partners 2. Identify effective interventions 3. Evaluate pilot results 4. Adapt pilot package for women Veterans; refine for new sites			1. Work with national-level partners 2. Review potential sites with partners			1. National-level partners send trainers for Gateway training at sites							1. Report on findings 2. Review business case 3. Collaborate to plan for spread 4. Collaborate with partners to re-tailor as needed with spread		

10. Identify and prepare champions				1. Site lead identify local CV Toolkit champion at each site 2. National-level partners travel to sites to train Gateway to Healthy Living facilitator			1. National-level partners travel to sites to train Gateway to Healthy Living facilitators									

11. Assess for readiness, & and identify barriers & and facilitators				1. Explore care options (health coaches, smoking cessation, MOVE!) at each of the sites during site visits 2. Conduct interviews and surveys with consenting key stakeholders												

12. Develop formal implementation blueprint							1. Further local tailoring with training and technical assistance of toolkit package for each site							1. Provide and elicit feedback to make modifications to implementation process to enhance local adoption and fidelity, and facilitate dissemination to future sites 2. Research team collaborate with local implementation teams to develop CV Toolkit Implementation Playbook								

13. Audit and provide feedback							1. Monitor and summarize use of computer template in deploying intervention in the clinic 2. Quarterly reports on use of computer template in deploying intervention presented to clinical teams							1. Provide and elicit feedback to make modifications to implementation process to enhance local adoption and fidelity, and facilitate dissemination to future sites		

14. Purposefully reexamine the implementation							1. Assess additional need for provider training 2. Quarterly reports on use of computer template in deploying intervention presented to clinical teams 3. Document in notes any issues with use of CV Toolkit during trainings in context of each clinic setting 4. Analyze notes in ATLAS.ti software in conjunction with evaluation data 5. Use monthly reflection calls during regular implementation meetings to assess and address implementation challenges							1. Provide and elicit feedback to make modifications to implementation process to enhance local adoption and fidelity, and facilitate dissemination to future sites		

15. Conduct cyclical small tests of change							1. Implement toolkit locally to ensure it works as intended with local systems and processes and make iterative changes as needed									

16. Develop an implementation glossary														1. Research team collaborate with local implementation teams to develop CV Toolkit Implementation Playbook		

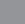
 *Develop stakeholder interrelationships*

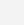
 *Train & and educate stakeholders*

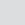
 *Use of evaluative and iterative strategies*

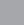
 *Adapt and tailor to context*

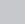
 *Provide interactive assistance*

**Table 3:** EMPOWER QUERI implementation evaluation: summary of methods.

Replicating Effective Programs phase*	Phase 1: pre-conditions						Phase 2: pre-implementation						Phase 3: implementation															Phase 4: maintenance and evolution			
				
Month	1	2	3	4	5	6	1	2	3	4	5	6	1	2	3	4	5	6	7	8	9	10	11	12	13	14	15	1	2	3	4
Provider and administrator interviews Phase 1: intervention planning, needs assessment, and acceptability; Phase 2: factors likely to affect adoption, acceptability, feasibility, satisfaction, penetration/reach. Phase 4: experiences of intervention/implementation; adaptations made in practice; suggestion for future adaptations to inform effectiveness and spread.				X						X																		X			

Provider surveys Measuring organizational readiness for patient engagement (more)										X																					

Patient interviews Phase 3: factors likely to affect adoption, acceptability, feasibility, satisfaction, penetration/reach. Phase 4: experiences of intervention/implementation; challenges, problem-solving, and suggestions for change/adaptation.													X															X			

Patient Surveys (pre- and post-intervention) *Primary outcomes*: program engagement and retention; change in targeted symptom or risk reduction behavior; *Secondary outcomes*: satisfaction (at f/u only), global health, out of role days; *Potential moderators*: engagement, patient demographics, social support, mental health													X												X						

Periodic reflections (discussions with team members to document) History and trajectory of implementation events Activities and interrelationships, including stakeholder engagement; Adaptations to intervention components and/or implementation strategies; Contextual factors with potential impact for implementation							X	X	X	X	X	X	X	X	X	X	X	X	X	X	X	X	X	X	X	X	X	X	X	X	X

Administrative data Referral monitoring Patient engagement Patient outcomes												X	X	X	X	X	X	X	X	X	X	X	X	X	X	X	X				

Text analysis Review of changes occurring to intervention components and/or implementation strategies per T1 (baseline) proposal materials and subsequent institutional review, amendments, and other study documentation																												X			

*At each implementation site, phases are expected to occur as follows: pre-conditions (6 months); pre-implementation (6 months); implementation (15 months): maintenance and evolution (4 months).

**Figure 2:**
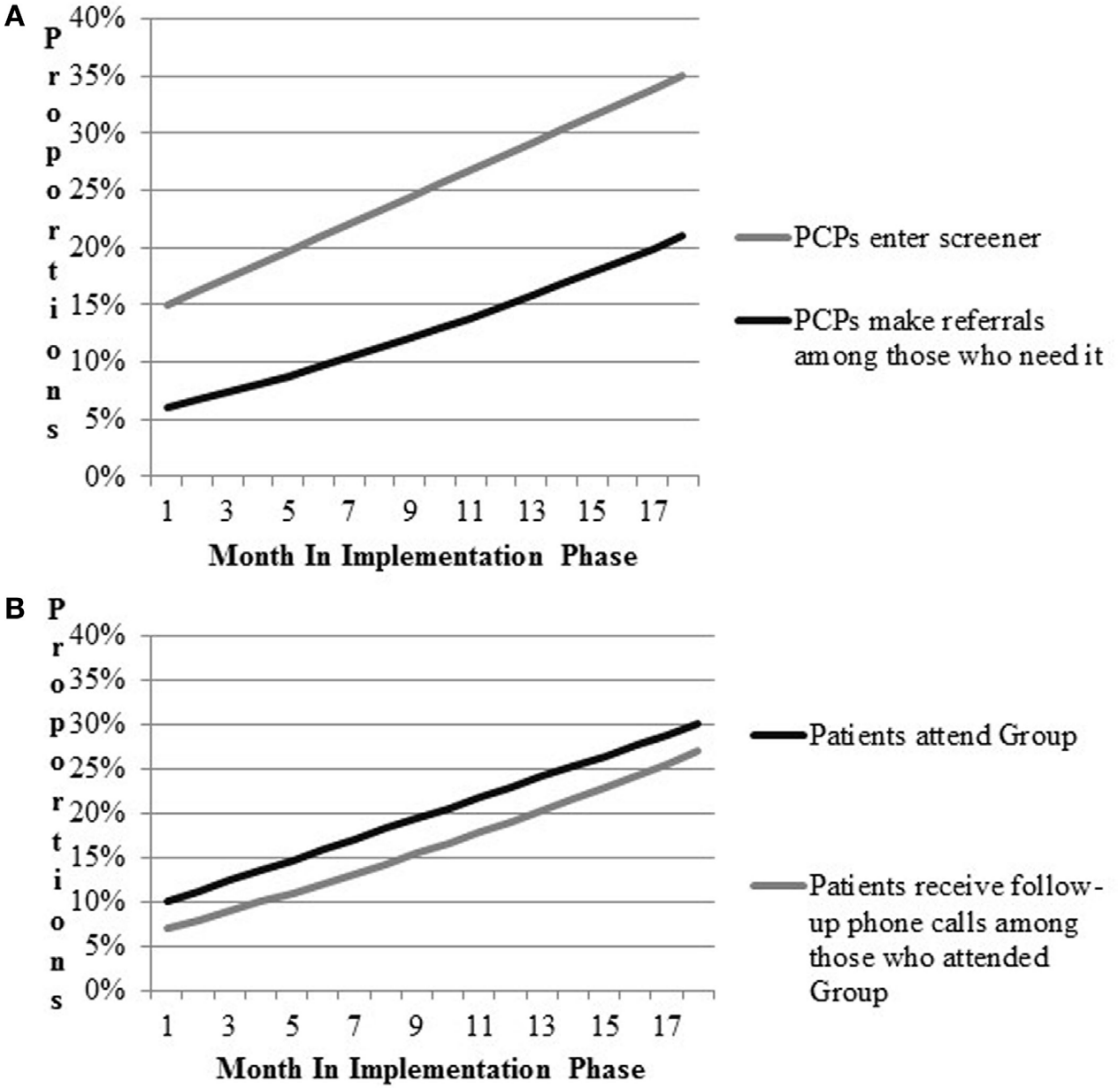
Prospective implementation scenario simulations for implementation outcomes. **(A)** CV Toolkit adoption. **(B)** Patient engagement.

As a means of verifying expected links between intervention components, implementation strategies, and outcomes of interest, we conducted a process of simulating data. Following the example of Zimmerman and colleagues (34), who suggest use of modeling to aid in implementation planning, we first mapped the flow of patients attending the women’s health primary care clinic and the process by which they receive referrals to the Gateway. Walking through the expected flow of patients in clinic with the study team, we estimated the likelihood of the provider completing the computer template, and making referral to Gateway; estimates were allowed a range of likelihood (e.g., 5–20%) to provide a lower and upper bound. We also estimated a rate of increase in these activities as the implementation period progressed. Estimates were intended to be conservative and were based in the team’s clinical and research experience of VA Women’s Health primary care clinics and change initiatives. Walking through the simulation process prompted useful discussion regarding where barriers and “bottlenecks” were likely to occur, stimulating discussion of how best to work with frontline providers and staff in overcoming those barriers. Final estimates were used to populate and refine a draft of a pragmatic implementation and evaluation blueprint that stipulates the general timing of activities and data collection, aids in assessing implementation outcomes, and ensures effective coordination of implementation and research activities (Figure 2). Strategy mapping activities occurred over the course of a one-year pre-implementation period during which other preparatory activities were ongoing, including identification of sites and site needs assessment and tailoring.

## Results

Table 4 below enumerates the 16 discrete implementation strategies intended for use as part of the CV Toolkit’s implementation effort according to enhanced REP. Strategies fell into five main categories, primarily related not only to use of evaluative and iterative strategies (6) and development of stakeholder interrelationships (5), but also reflecting efforts to train and educate stakeholders (2), adapt and tailor to context (2), and provide interactive assistance (1).

**Table 4:** Number of implementation strategies by conceptual cluster (10).

Strategy conceptual cluster	Frequency	Strategy
Develop stakeholder interrelationships	5	Involve executive boards
		Build a coalition
		Inform local opinion leaders
		Identify and prepare champions
		Develop an implementation glossary

Use evaluative and iterative strategies	6	Conduct local needs assessment
		Conduct cyclical small tests of change
		Assess for readiness and identify barriers and facilitators (local resources)
		Develop formal implementation blueprint
		Audit and provide feedback
		Purposely reexamine the implementation

Train and educate stakeholders	2	Conduct educational meetings
		Develop educational materials

Adapt and tailor to context	2	Tailor strategies
		Promote adaptability

Provide interactive assistance	1	Provide local technical assistance

Table 3 delineates planned use of strategies across each of the four REP phases. Four of the 16 strategies identified are to be deployed during a single REP phase: conduct local needs assessment in the pre-condition phase; assess for readiness and identify barriers and facilitators in pre-implementation; conduct cyclical small tests of change during implementation; and develop an implementation glossary during maintenance and evolution. All other strategies occurred across more than one phase of the implementation effort.

Most (9 out of 16) strategies are initiated in the pre-condition phase. These nine are varied and include the following: involve executive boards; build a coalition; inform local opinion leaders; conduct local needs assessment; develop educational materials; conduct educational meetings; tailor strategies; promote adaptability; and provide local technical assistance. By contrast, there are fewer implementation strategies initiated in the remaining REP phases: two in the REP pre-implementation phase (identify and prepare champions and assess for readiness and identify barriers and facilitators), four in the implementation phase (conduct cyclical small tests of change, develop formal implementation blueprint, audit and provide feedback, and purposely reexamine the implementation), and one in the maintenance and evolution phase (develop an implementation glossary). Strategies occurring in later REP phases focus on two main categories of activity: use of evaluative and iterative strategies and developing stakeholder interrelationships.

Once initiated, most strategies (12 of the 16) are to be deployed during multiple REP phases. For example, strategies that involve training and education of stakeholders (e.g., developing educational materials and conducting educational meetings) are deployed during pre-condition, pre-implementation, and implementation phases, as are strategies for informing local opinion leaders, providing local technical assistance, and identifying and preparing champions. Most strategies that involve use of evaluative and iterative strategies (e.g., developing formal implementation blueprint, audit and provide feedback, and purposely reexamine the implementation) are to be deployed during implementation and maintenance and evolution phases. Strategies for promoting adaptability are deployed during the latter three REP phases (pre-implementation, implementation, and maintenance and evolution), while strategies for building a coalition occur across pre-conditions, pre-implementation, and maintenance and evaluation phases. Finally, two of the strategies (tailor strategies and involve executive boards) are deployed during all four REP phases.

Results for the implementation scenario simulations are presented in Figure 2. Figure 2A includes outcomes related to providers’ entry of CV risk screener data into the medical record and referrals to VA programs. Figure 2B models attendance at Gateway groups and follow-up phone calls to Gateway participants. Team members hypothesized that providers would enter patient screener information into the CV template during patient appointments 15% of the time during early implementation. Team members expected improvements in the proportions of providers entering the information over time, such that at the end of 18 months of implementation, the proportion would increase to 35%. Second, team members hypothesized that referrals by providers to other VA services would increase by 15% by the end of implementation. Based on these parameters, approximately up to 21% of patients were expected to be receiving any new referrals by the end of the study period. Team members hypothesized that Gateway participation would increase to 30% and most participants would receive follow-up phone calls by the end of implementation.

## Discussion

Recent guidelines for specifying implementation strategies raise challenges for implementation efforts making use of multiple or packaged strategies, such as the use of enhanced REP in the EMPOWER QUERI. These challenges include how best to describe each individual strategy and its components, develop a practical blueprint for operationalizing implementation and research activities, and ultimately, plan for a program evaluation that takes the cumulative impact of packaged strategies into account. We conducted a prospective, formative, and iterative process of strategy mapping to address these challenges, mapping implementation activities and strategies into an explicit blueprint by implementation phase and conducting a simulation exercise with project team members to validate our evaluation plan. The blueprint articulates the projections of what we anticipate in implementing the CV Toolkit, and serves as an accounting tool that allows us to track and compare our projections to on-the-ground implementation progress as we carry out the intervention. The method of mapping has provided new insight into where, when, and how each strategy is deployed, allowing us to formulate a targeted multi-method evaluation plan.

We identified five categories of strategies to be used in the implementation of the CV Toolkit: use of evaluative and iterative strategies, develop stakeholder interrelationships, adapt and tailor to context, train and educate stakeholders, and provide interactive assistance. These five categories correspond to the five that Waltz and colleagues rated as having the highest importance in achieving successful implementation (10). Communication, an additional category of strategies suggested by Boyd and colleagues (13), appeared to emerge in these data as an essential component of nearly all strategies, rather than a distinct category unto itself. We also mapped evaluative and iterative strategies as occurring most frequently in the CV Toolkit implementation, an emphasis that appears to be supported by Waltz and colleagues’ rating of evaluative and iterative strategies as the single most important category of strategies. It is noteworthy that explicitly financial strategies are not used in the CV Toolkit. This contrasts with the work of Honeycutt and colleagues, who identified financial and technical assistance as effective mechanisms for dissemination of evidence-based programs (35). Similarly, Cunningham and Card found that funding, staff, and other resources was the only factor significantly associated with implementation of evidence-based interventions (17). In future work, it will be important to compare how financial strategies affect implementation in integrated versus decentralized healthcare systems (36).

In addition to identifying the relative frequency of strategies, mapping the list of discrete strategies to be used across REP phases provided significant insight into the timing of when strategies are used in this project, and to what ends. For example, although evaluative and iterative strategies are the most frequently occurring, these strategies occur primarily during implementation and maintenance and evolution phases. By contrast, most other strategies are initiated in the pre-conditions phase, thus underscoring the importance of the early phase in laying the groundwork for large-scale implementation studies. Our current study is similar to other implementation evaluation studies that examine implementation by phases, such as that by Chamberlain and colleagues, who focused on two implementation strategies and found that sites ceased progress during pre-implementation phase (15). Similarly, Blackford and colleagues (19) have also made use of an evaluation tool to track progress in implementing an advance care planning initiative, finding the tool useful in supporting planning, tracking progress, and providing direction for future change. In all, our current study and those in the literature speak to the importance of timing in evaluating how differing strategies support effective implementation.

We found dose to be the most difficult domain to define for 12 of the 16 strategies mapped, and specifically for those strategies deployed across multiple REP phases. Issues to be resolved include how to quantify dose for each strategy (e.g., unit of analysis), the relationship between length of time and intensity of effort involved in calculating dose, and what activities “count” as deployment of a strategy, e.g., if a strategy is used only briefly or mentioned in an email. Additional issues that arose include how best to quantify the cumulative effects of strategies deployed at multiple phases, e.g., additively or multiplicatively. These issues hold true for all strategies except for the four that we identified as being deployed during a single REP phase, which are more easily counted and tracked as activities. In pragmatic implementation, it may not always be feasible or practical to specify every component of implementation strategies when working with complex, multi-component packages. The literature points to differing approaches as to how to define dosage in implementation evaluation studies. For example, Boyd and colleagues operationalized dose as intent to use strategies (13)*. Similarly, Ferm and colleagues defined dose in terms of intervention fidelity (i.e., number of sessions of the intervention compared to the number of sessions that was supposed to be delivered). By contrast, Bunger and colleagues* (11) operationalized dose in terms of person-hours invested in implementation. Honeycutt and colleagues (35) found that sites implementing had different interpretations of defining completion of core elements and suggested that future studies might benefit from explicit guidance on quantifying dose of program core elements. Nonetheless, the recent guidelines by Powell, Proctor and colleagues encourage thoughtful attention to these components.

Simulating the implementation scenarios in which the CV Toolkit is deployed was helpful because it served as a “run-through” of our evaluation plan. We identified the many moving and interacting components of the Toolkit and how each is likely to contribute to the outcomes of interest. We also clarified the information that we can expect to collect routinely *over time* and across sites, which we expect to serve as parameters and data for longitudinal analyses. The simulation exercise also served to validate our evaluation plan that explicitly accounts for the multi-level structure of the data, taking into consideration the context-dependent nature of implementing the Toolkit.

We believe there are a number of advantages to the strategy mapping approach described here. This method provides a low-burden process for achieving specification of strategies. It also supports developing an implementation blueprint and comprehensive evaluation plan, with potential for examining adherence. We also believe that strategy mapping is likely to be easier and more supportive of effective implementation if done prospectively rather than retrospectively. Mapping is likely to be fruitful in ensuring that all elements of an implementation research effort—including the intervention, implementation plan, and evaluation plan—have been clearly articulated prior to launch. In the case of the CV Toolkit project, the mapping process has provided structure for implementation by allowing for detailed front-end specification of project activities, development of a succinct but comprehensive blueprint for activities across each of the four REP phases, and simulation of the longitudinal quantitative data likely to emerge across sites, thus providing both guidelines for and an opportunity to “test-run” implementation and evaluation activities. Visual representation of planned strategy rollout can also serve as a tracking tool to support identifying where the project, or a specific site, deviates from the expected use of or sequencing of strategies. Mapping strategies helps to organize, plan, and clarify the implementation process by specifying the necessary action steps per phase, and milestones along the implementation timeline. Moreover, mapping implementation strategies allows us to identify and prioritize key strategies that we can leverage to improve outcomes. Finally, as we move forward with CV Toolkit implementation, in partnership with local and national stakeholders, we expect that strategy mapping will also support development of implementation playbooks (37)—i.e., brief primers providing “how to” or “lessons learned” information—intended to facilitate more rapid dissemination, scale-up, and spread.

Potential disadvantages of this approach include the fact that it requires substantial time during the initial project planning phases. We conducted the activities described over a one-year period preparatory to implementation launch; however, we believe this process could be conducted much more rapidly following the outline offered here. Although mapping strategies across multiple phases of implementation requires some thought and attention a priori, our process is relatively low burden, and no more intensive than the detailed logs of implementation activities used in other approaches (11, 13). Another disadvantage may be that this mapping approach requires additional tracking to document whether strategies are ultimately implemented as planned or whether the plan is adapted as implementation proceeds. However, we believe that strategy mapping preparatory to implementation is likely to make tracking easier and potentially more accurate by functioning as a practical checklist for expected activities that allows for the benchmarking of implementation progress.

Future research should continue to explore the utility of this and other methods for mapping strategies in complex implementation. One interesting possibility for this work is likely to involve a more participatory approach, working directly with sites and other stakeholders to delineate key strategies and plan for pragmatic evaluation. The role of data capture in providing information on whether and when adoption is occurring provides the opportunity to further explore how best to observe, track, and communicate with stakeholders regarding implementation progress and outcomes (38). We are continuing to explore questions related to the analytic utility of strategy mapping as we proceed with the multi-site CV Toolkit study, including whether the process can be used to identify core components of packaged strategies like our enhanced REP, whether specific categories of strategies appear to be associated with specific outcomes [similar to the approach used by Boyd et al. (13)], and whether differing combinations or sequences of strategies appear to be associated with differential outcomes [similar to the findings by Yakovchenko (18)]. Notably, as illustrated in Table 3, our evaluation plan is multi-method and integrates both quantitative and qualitative data sources to address these research questions. For example, in addition to the questions related to adoption and reach of the CV Toolkit examined directly in the simulation exercise described above, we are also using semi-structured interviews to assess acceptability, feasibility, and satisfaction among patients receiving the CV Toolkit and providers and staff members delivering the CV Toolkit in their clinics.

## Conclusion

We update recent guidance on specification of implementation strategies by considering the implications of such guidance for use of multi-strategy frameworks such as enhanced REP, and propose a novel method to support strategy mapping in complex interventions, with the goal of facilitating both implementation and evaluation efforts. Our strategy mapping approach is innovative in offering a clear and structured method for stipulating when and how implementation strategies occur across the entire life cycle of an implementation effort, in this case across the four REP phases. By doing so, the method aids in fully documenting how implementation activities proceed, to support more effective description and replicability where implementation proves successful. This method also aids in developing plans for evaluation and analysis by clarifying the timing of events and where specific implementation strategies are occurring singly or in combination. Our results identified interesting patterns in the sequence of strategies, particularly related to the importance of pre-implementation activities in laying the groundwork for implementation, as well as the differing ways that specific implementations strategies may be used across different REP phases (e.g., with coalition partners providing support for local uptake during early phases and informing strategies for dissemination and spread in later phases). This approach may therefore be of particular usefulness in implementation efforts employing multi-phase frameworks, such as EPIS (23). Ultimately, understanding timing of implementation strategies will aid in the summative evaluation that utilizes the non-randomized stepped wedge design that explicitly accommodates for the naturalistic roll-out of interventions and programs. Furthermore, specifying strategies into their functional components provides a level of detail on implementation activities that is likely to aid in identifying not only whether the overall implementation has been successful in impacting clinical and patient outcomes, but also by what mechanisms. Finally, in operationalizing and specifying the implementation strategies used in each phase of implementation, we seek to advance understanding of how implementation strategies—individually and in combination—function to support effective practice change. The work presented here provides a model for developing comprehensive implementation and evaluation blueprints to support the increasing methodological complexity of work being done in implementation science.

## Author Contributions

AKH developed the method, analyzed, synthesized, and interpreted the findings, and drafted and critically revised the manuscript. ABH conceived the design of the overall project and manuscript, provided feedback on the method, interpretations of implementation, and research activities, interpreted the findings, and drafted and critically revised the manuscript. BB-M provided feedback on the method, interpretations of implementation, and research activities, interpreted the findings, and drafted and critically revised the manuscript. MF provided feedback on the method, interpretations of implementation, and research activities, interpreted the findings, and drafted and critically revised the manuscript. SS provided feedback and interpretations of implementation and research activities, interpreted the findings, and drafted and critically revised the manuscript. TM provided feedback and interpretations of implementation and research activities, interpreted the findings, and drafted and critically revised the manuscript. EF developed the method, analyzed, synthesized, and interpreted the findings, and drafted and critically revised the manuscript.

## Conflict of Interest Statement

The authors declare that the research was conducted in the absence of any commercial or financial relationships that could be construed as a potential conflict of interest.
